# Time to pregnancy in women with previous ectopic pregnancy undergoing in vitro fertilization treatment: a retrospective cohort study

**DOI:** 10.1038/s41598-022-13027-1

**Published:** 2022-05-25

**Authors:** Yamei Xue, Fuxing Zhang, Haocheng Zhang, Songying Zhang

**Affiliations:** Assisted Reproduction Unit, Department of Obstetrics and Gynecology, Key Laboratory of Reproductive Dysfunction Management of Zhejiang Province, Sir Run Run Shaw Hospital, School of Medicine, Zhejiang University, 3 Qingchun East Rd., Hangzhou, 310016 Zhejiang Province China

**Keywords:** Infertility, Reproductive disorders

## Abstract

We aimed to investigate the difference in the time to pregnancy (TTP) between women with previous ectopic pregnancy (EP) and control women following in vitro fertilization (IVF) treatment and the association between TTP and the number of oocytes retrieved and embryos available. A retrospective study involving 1097 women, 547 of which had previous EP and 550 were control women whose previous pregnancy were abortion, was conducted. Women in the EP group had significantly longer median TTP than those in the control group (36; range, 12–252 vs 28; range, 12–220; P = 0.019). For women with previous EP, > 48 months TTP was most likely associated with low numbers of oocytes retrieved and embryos available compared to TTP of ≤ 24 months or 25–48 months, and women with younger age had a shorter TTP, higher numbers of oocytes retrieved and embryos available. A Cox proportional hazards model showed that maternal age was significantly related to the pregnancy over the TTP (adjusted hazard ratio, 0.934; P < 0.001). In conclusion, women with previous EP have a significantly increased TTP than control women with previous abortion. For women with previous EP, TTP is negatively associated with the numbers of oocytes retrieved and embryos available.

## Introduction

Approximately 2% of spontaneous pregnancies are ectopic^1–3^. Ectopic pregnancy (EP) happens when the embryo implants outside the uterine cavity, mostly in the fallopian tubes^1–3^. Tubal EP remains the most common cause of pregnancy-related mortality in the first trimester of pregnancy^4^. In recent years, early diagnosis has made the maternal death caused by EP more and more rare and the clinical emphasis has focused on optimizing treatment methods to preservation of fertility^5–7^ or on the ability of women to obtain intrauterine pregnancy after EP^3,8,9^.

Several studies have reported that the women with previous EP had a reduced fecundability. Early literature found that nearly 50% of patients after one tubal pregnancy will be involuntarily infertile^10^. A population-based cohort study reported that women with ectopic first pregnancy were 19% less likely to have an intrauterine second pregnancy compared with women whose first pregnancy was intrauterine^3^. In a larger study assessing long-term reproductive prognosis in women whose first is EP, Lund Kårhus L et al. found these women had the lowest delivery rate compared with women with a first miscarriage or a first induced abortion and concluded that fertility is compromised in women whose first pregnancy is EP^11^, which is consistent with the results of another published data^12^.

Although some studies have reported natural pregnancy outcomes at 2, 6, and 15 years after the initial EP^11,13,14^, most patients do not consider 6 years or more a reasonable amount of time to achieve pregnancy. The urgency to conceive often leads these patients to consider in vitro fertilization (IVF) treatment to increase their chance of a pregnancy sooner, as well as reduce the time to their next pregnancy. Possible benefits of undergoing fertility treatments are an increased number of mature oocytes available per cycle and improved timing of fertilization.

When counseling these infertile women, consideration of reproductive potential following EP is important. Infertile women are concerned about their subsequent pregnancy outcomes after IVF treatment. Most women desire a more specific time line on how long it typically takes for a fertile patient with previous EP to get pregnant after IVF treatment. However, due to the lack of robust data, consultation is always challenging for doctors.

Time to pregnancy (TTP), defined as the time between the start of attempts to become pregnant and reaching an actual pregnancy, has been regarded as a useful tool for the evaluation of reproductive effect^15–17^. To examine the existing evidence about the TTP in women with previous EP, we conducted a literature search in Ovid MEDLINE (1966–2021) and EMBASE (1996–2021) using the search terms “ectopic”, “pregnancy or gestation”, and “time to pregnancy”. Only very few studies investigated TTP in women with previous EP^12,18^. Two studies addressing this issue reported contradictive results^12,18^. Hassan and Killick found that the reduced fecundity was observed in women after EP and adjusted mean TTP after EP was 1.8-fold longer compared with TTP after the termination of pregnancy, although the sample size of women with previous EP is too small^18^. A much larger study reported that women with previous EP had an increased chance of second pregnancy within two years of TTP compared to women with previous abortion^12^.

Given the limited evidence and inconsistent results, the aim of this present study was carried out: (1) to verify whether differences occur in the TTP between pregnant women following IVF treatment whose first pregnancy is ectopic and women whose first pregnancy is abortion; (2) to evaluate whether short and long TTP was associated with the number of oocytes retrieved and embryos available; (3) to investigate the association between TTP and the number of oocytes retrieved and embryos available among the women stratified by maternal age.

## Methods

### Patients

We performed this single-center, retrospective study at the Reproductive Medicine Center, Sir Run Run Shaw Hospital, College of Medicine, Zhejiang University, and collected intrauterine pregnant patients with a history of tubal EP or abortion after the first IVF-ET treatment from January 2016 to January 2020. Intrauterine pregnancy was defined as the presence of an intrauterine gestational sac with a heartbeat on ultrasound. Abortion was defined as the spontaneous or induced termination of pregnancy.

Inclusion criteria were women with (1) achieving an intrauterine pregnancy following the first IVF treatment, (2) a history of tubal EP or abortion from a previous natural pregnancy, (3) age between 20 and 45 years old at the time of the commencement of IVF treatment, and (4) regular menstrual cycle (interval 21–35 days). Exclusion criteria were as follow: (1) pregnancy resulted from donor oocytes or preimplantation genetic diagnosis/screening, (2) women who had changed partners since the prior EP or abortion, (3) women who had repeated abortion, and (4) no complete record. The size of the total study population in the present study was 1097 pregnant women, including 547 women who had an EP in their previous natural pregnancy (the EP group) and 550 women who had an abortion in their previous pregnancy (the control group).

Patients were identified in the electronic medical record via retrospective review of all IVF-ET cycles over the study period. Collected data included maternal age, mother’s body mass index (BMI) before the pregnancy, mother’s education, basal serum FSH, LH, E2, antral follicle count (AFC), cause of infertility, stimulation protocols, duration of stimulation, number of oocytes retrieved, fertilization method, the number of embryos available, number of embryos transferred, stage of embryos transferred (cleavage embryo or blastocyst). TTP was defined as the interval between the start of attempts to become pregnant after previous tubal EP or abortion and the observation of an actual pregnancy following IVF treatment.

### Clinical and laboratory protocols

Controlled ovarian hyperstimulation (COH) was performed as previously described^19^. COH was carried out to maximize follicular response while minimizing the risk of ovarian hyperstimulation syndrome. Gonadotropin doses (Gonal-F, Serono Laboratories, Aubonne, Switzerland; or Puregon, N.V. Organon, Oss, the Netherlands) were adjusted according to women's age, weight, antral follicle count, and follicular response to stimulation, if any. Final oocyte maturation was induced using human chorionic gonadotropin (hCG) (6500–10,000 IU; Serono Laboratories, Modugno, Italy) when at least three or more follicles (mean diameter of 16–18 mm or more) were present.

Oocyte retrieval was performed 35–37 h after hCG administration by transvaginal ultrasonography-guided aspiration. Fertilization was performed using either conventional IVF or intracytoplasmic sperm injection (ICSI). Embryos were cultured with the use of sequential media, and embryonic development was assessed on day 3 after oocyte retrieval. Embryo morphology was scored as: grade 1, equal blastomeres with no obvious fragmentation; grade 2, < 20% fragmentation and/or unequal blastomeres; grade 3, 20–50% cytoplasm fragmentation; grade 4, > 50% cytoplasm fragmentation. Embryos available in this study referred to the grade 1 to 3 embryos on day 3. Fresh embryo transfers were performed on day 3, day 5, or day 6 after oocyte retrieval. The number of embryos or blastocysts transferred was in line with the guideline of the Fourth Session of the Committee of Chinese Society of Reproductive Medicine (CSRM)^20^.

Embryo transfer was performed using a soft catheter (Sydney, Cook, Melbourne, Australia) under transabdominal ultrasound guidance by the senior physicians with a minimum of 5 years of transferring experience at our center. Patients were asked to keep a full bladder to facilitate an ultrasound view of the uterine cavity. Before ET, the cervical mucus was gently removed using sterile cotton swabs as needed. The embryo(s) was/were transferred about 1 to 1.5 cm from the uterine fundus under ultrasound visualization. After transfer, the catheter was immediately and carefully checked for retained embryos and the presence of blood under a microscope.

### Statistical analysis

All statistical analyses were performed with the use of Statistical Package for Social Sciences version 20.0 (SPSS, Chicago, IL, USA). For continuous variables, we present mean and standard deviation (SD) for symmetrical distribution or median and range (minimum–maximum values) for asymmetrical distributions. The variables were compared with one way ANOVA test or the nonparametric Mann–Whitney test depending on whether the data were normally distributed. Categoric variables were compared with Pearson’s Chi-squared or Fisher's exact test according to the sample size. The Cox proportional hazards regression model was performed to examine the hazard ratio with 95% confidence intervals to evaluate the differences across various parameters. The likelihood of pregnancy was determined by using the log-rank (Mantel-Cox) test applied to the Kaplan Meier survival curves built up for each group. The result was considered statistical significance if the P-value was < 0.05.

### Ethical approval

The study was approved by the Reproductive Medical Ethics Committee of Sir Run Run Shaw Hospital. Informed consent was obtained from all the patients. And this study complied with declaration of Helsinki/relevant guidelines for the study on humans.

## Results

Overall, 1097 women—547 women with previous EP (EP group) and 550 women with previous abortion (Control group)—were included in the study. Cohort characteristics are displayed in Table 1. The median age was 33 years (range 22–45 years) and 31 years (range 20–45 years) for the EP group and the control group (P < 0.001), respectively. Women in the EP group had a significantly lower median AFC than those in the control group (7; range, 1–25 vs 9; range, 1–26; P < 0.001). There was a greater proportion of couples with tubal and male factors infertility in the EP group compared with couples in the control group. The infertility factors of diminished ovarian reserve and endometrial-uterine were similar between the two groups. Women in the EP group had significantly lower median numbers of oocytes retrieved (7; range, 1–35 vs 11; range, 1–39; P < 0.001) and embryos available (5; range, 1–21 vs 6; range, 1–29; P < 0.001) than those in the control group. The median TTP in the EP group and control group was 36 months (range 12–252) and 28 months (range 12–220), respectively (P = 0.019). There was no significant difference in BMI, level of education, basal serum FSH, LH, and E2 levels, the proportion of ovarian stimulation protocols, the duration of stimulation, methods of fertilization, two pronucleus rate, stage of embryos transferred, and the number of embryos transferred between the two groups.

**Table 1:** Main demographics and cycle characteristics of patients.

Characteristics	EP group	Control group	P value
	N = 547	N = 550	
Maternal age (median and range)	33 (22–45)	31 (20–45)	< 0.001
BMI (kg/m^2^) (mean ± SD)	21.1 ± 2.4	21.2 ± 2.5	0.752
**Level of education (%)**			
Low	160 (29.3)	133 (24.2)	0.058
Middle	236 (43.1)	240 (43.6)	0.869
High	151 (27.6)	177 (32.2)	0.098
Basal serum FSH (IU/L) (mean ± SD)	8.1 ± 3.2	7.8 ± 3.0	0.669
Basal serum LH (IU/L) (mean ± SD)	4.5 ± 2.4	4.8 ± 3.6	0.629
Basal serum E2 (ng/L) (mean ± SD)	37.4 ± 21.5	40.0 ± 25.2	0.658
AFC (median and range)	7 (1–25)	9 (1–26)	< 0.001
**Cause of infertility (%)**			
Tubal factor	547 (100)	309 (56.2)	< 0.001
Diminished ovarian reserve	59 (10.7)	61 (11.1)	0.872
Endometrial-uterine factor	49 (9.0)	52 (9.5)	0.776
Male factor	110 (20.1)	162 (29.5)	< 0.001
**Stimulation protocol (%)**			0.379
Long protocol	282 (51.6)	293 (53.3)	
Short protocol	104 (19.0)	93 (16.9)	
Micro-stimulation protocol	91 (16.6)	106 (19.3)	
Others	70 (12.8)	58 (10.5)	
Duration of stimulation (day) (mean ± SD)	8.6 ± 2.6	8.3 ± 2.9	0.091
No. of oocytes retrieved (median and range)	7 (1–35)	11 (1–39)	< 0.001
**Methods of fertilization (%)**			
IVF	428 (78.2)	412 (74.9)	0.192
ICSI	119 (21.8)	138 (25.1)	
Two pronucleus rate (median and range)	0.7 (0.1–1)	0.7 (0.1–1)	0.362
No. of embryos available (median and range)	5 (1–21)	6 (1–29)	< 0.001
**Stage of embryos transferred (%)**			
Cleavage	361 (66.0)	389 (70.7)	0.092
Blastocyst	186 (34.0)	161 (29.3)	
**No. of embryos transferred (%)**			
1	180 (32.9)	204 (37.1)	0.146
2	360 (65.8)	340 (61.8)	0.169
3	7 (1.3)	6 (1.1)	0.773
Time to pregnancy (m) (median and range)	36 (12–252)	28 (12–220)	0.019

*EP* ectopic pregnancy, *BMI* body mass index, *AFC* antral follicle count, *FSH* follicle-stimulating hormone, *LH* luteinizing hormone, *SD* standard deviation, *IVF* in vitro fertilization, *ICSI* intracytoplasmic sperm injection.

Table 2 compares the maternal age, the number of oocytes retrieved, and embryos available of both two groups, stratified by time to pregnancy. In the EP group, the median age was generally younger in women with shorter TTP: 31 years (range 22–45) in women with ≤ 24-month TTP, 33 years (range 23–45) in women with 25- to 48-month TTP, 35 years (range 25–45) in women with ≥ 49-month TTP. The median number of oocytes retrieved was generally lower in longer TTP: 5 (1–22) in ≥ 49-month TTP, 7 (1–29) in 25- to 48-month TTP, 9 (1–35) in ≤ 24-month TTP. The number of embryos available was significantly lower in the ≥ 49-month TTP group (median: 4, range: 1–15) compared with those in the 25- to 48-month TTP group (median: 5, range: 1–21) and in the ≤ 24-month TTP (median: 5, range: 1–19). However, in the control group, there was no significant difference in the median age, the number of oocytes retrieved, and embryos available between the different TTP. Between the EP group and control group, the median age, the number of oocytes retrieved, and the number of embryos available among women with 25- to 48-month TTP and ≥ 49-month TTP exist significant differences.

**Table 2:** Maternal age, the number of oocytes retrieved and embryos available in both two groups, stratified by time to pregnancy.

	Time to pregnancy, months			P value^1^		
	≤ 24	25–48	≥ 49	P1	P2	P3
**EP group**						
N (%)	252 (46.1)	163 (29.8)	132 (24.1)	–	–	–
Maternal age (median and range)	31 (22–45)	33 (23–45)	35 (25–45)	0.005	< 0.001	< 0.001
No. of oocytes retrieved (median and range)	9 (1–35)	7 (1–29)	5 (1–22)	0.002	< 0.001	< 0.001
No. of embryos available (median and range)	5 (1–19)	5 (1–21)	4 (1–15)	0.091	< 0.001	< 0.001
**Control group**						
N (%)	258 (46.9)	223 (40.5)	69 (12.5)	–	–	–
Maternal age (median and range)	32 (20–43)	31 (23–44)	31 (23–45)	0.992	0.377	0.402
No. of oocytes retrieved (median and range)	10 (1–29)	8 (1–39)	7 (2–27)	0.085	0.585	0.463
No. of embryos available (median and range)	6 (1–18)	6 (1–29)	6 (1–17)	0.206	0.991	0.470
P value defined as the comparison in maternal age between EP and control group	0.435	0.029	< 0.001			
P value defined as the comparison in number of oocytes retrieved between EP and control group	0.013	< 0.001	< 0.001			
P value defined as the comparison in number of embryos available between EP and control group	0.202	< 0.001	< 0.001			

^1^We defined the statistical outcomes of ≤ 24 months group versus 25–48 months group as P1; ≤ 24 months group versus ≥ 49 months group as P2; 25–48 months group versus ≥ 61 months group as P3. *IVF* in vitro fertilization, *EP* ectopic pregnancy.

A Cox proportional hazards model was fitted to determine the influence of several parameters on TTP to intrauterine pregnancy in patients with previous EP. The results are shown in Table 3. After adjusting maternal age, level of education, AFC, cause of infertility, duration of stimulation, the number of oocytes retrieved, the number of embryos available, stage of embryos transferred, the number of embryos transferred in the model, maternal age was significantly related to the intrauterine pregnancy over the TTP (adjusted hazard ratio, 0.934; P < 0.001).

**Table 3:** Hazard ratio for pregnancy per time to pregnancy in patients with previous ectopic pregnancy.

	Unadjusted (95% CI)	P value	Adjusted (95% CI)	P value
Maternal age	0.971 (0.953–0.989)	0.002	0.934 (0.914–0.954)	< 0.001
No. of oocytes retrieved	0.994 (0.980–1.009)	0.442	1.010 (0.973–1.049)	0.591
No. of embryos available	0.993 (0.970–1.016)	0.547	1.010 (0.965–1.057)	0.678
Stage of embryos transferred	0.384 (0.327–0.452)	< 0.001	1.068 (0.867–1.315)	0.534

In order to verify whether the maternal age of study women could be involved in TTP, we compared the outcome of different age groups. The detailed data are reported in Table 4 and show that women with older age had a longer TTP, lower numbers of oocytes retrieved, and embryos available.

**Table 4:** Time to pregnancy, the number of oocytes retrieved and embryos available in patients with previous ectopic pregnancy, stratified by maternal age.

	Maternal age, years			P value^1^		
	< 30	30 to < 35	≥ 35	P1	P2	P3
	N = 147	N = 208	N = 192			
Time to pregnancy (month) (median and range)	24 (12–84)	30 (12–144)	36 (12–252)	0.001	< 0.001	< 0.001
No. of oocytes retrieved (median and range)	11 (1–29)	8 (1–35)	5 (1–29)	< 0.001	< 0.001	< 0.001
No. of embryos available (median and range)	6 (1–19)	5 (1–19)	4 (1–21)	0.028	< 0.001	< 0.001

^1^We defined the statistical outcomes of < 30 years group versus 30 to < 35 years group as P1; < 30 years group versus ≥ 35 years group as P2; and 30 to < 35 years group versus ≥ 35 years group as P3.

To evaluate the effect of different treatments of previous ectopic pregnancy on the IVF outcomes, the patients were categorized into three groups: salpingectomy group, salpingostomy group, and conservative treatment group. There was no significant difference in maternal age, basal serum FSH, LH, E2, AFC, stimulation protocols, and duration of stimulation among the three groups. The number of oocytes retrieved, two pronucleus rates and the number of embryos available also did not differ. The results are shown in Table 5.

**Table 5:** Cycle characteristics of patients with previous ectopic pregnancy according to treatments.

Characteristics	Salpingectomy N = 300	Salpingostomy N = 155	Conservative treatment N = 92	P-value^1^		
				P1	P2	P3
Maternal age (median and range)	33 (22–45)	33(23–45)	33(23–42)	0.633	0.924	0.673
Basal serum FSH (IU/L) (mean ± SD)	8.3 ± 3.2	7.9 ± 3.3	8.7 ± 3.8	0.062	0.405	0.051
Basal serum LH (IU/L) (mean ± SD)	4.3 ± 1.9	4.8 ± 3.2	4.7 ± 2.6	0.200	0.676	0.566
Basal serum E2 (ng/L) (mean ± SD)	37.3 ± 21.8	40.0 ± 25.3	35.5 ± 22.8	0.310	0.579	0.212
AFC (median and range)	7 (2–21)	6 (2–22)	6 (1–25)	0.415	0.853	0.665
**Stimulation protocol (%)**				0.171	0.290	0.556
Long	139 (46.3)	81 (52.3)	53 (57.6)			
Short	68 (22.7)	22 (14.2)	16 (17.4)			
Micro-stimulation	53 (17.7)	27 (17.4)	12 (13.0)			
Others	40 (13.3)	25 (16.1)	11 (12.0)			
Duration of stimulation (d) (mean ± SD)	8.5 ± 2.7	8.7 ± 2.6	8.8 ± 2.4	0.196	0.246	0.909
No. of oocytes retrieved (median and range)	6 (1–29)	7 (1–35)	7(1–27)	0.733	0.500	0.707
**Methods of fertilization (%)**				0.374	0.961	0.469
IVF	229 (76.3)	124 (80.0)	70 (76.1)			
ICSI	71 (23.7)	31 (20.0)	22 (23.9)			
Two pronucleus rate (median and range)	0.7 (0.1–1)	0.7 (0.1–1)	0.7 (0.1–1)	0.828	0.682	0.813
No. of embryos available (median and range)	4 (1–29)	4 (1–25)	4 (1–22)	0.563	0.336	0.723

^1^We defined the statistical outcomes of salpingectomy group versus salpingostomy group as P1; salpingectomy group versus conservative treatment group as P2; salpingostomy group versus conservative treatment group as P3.

**Figure 1:**
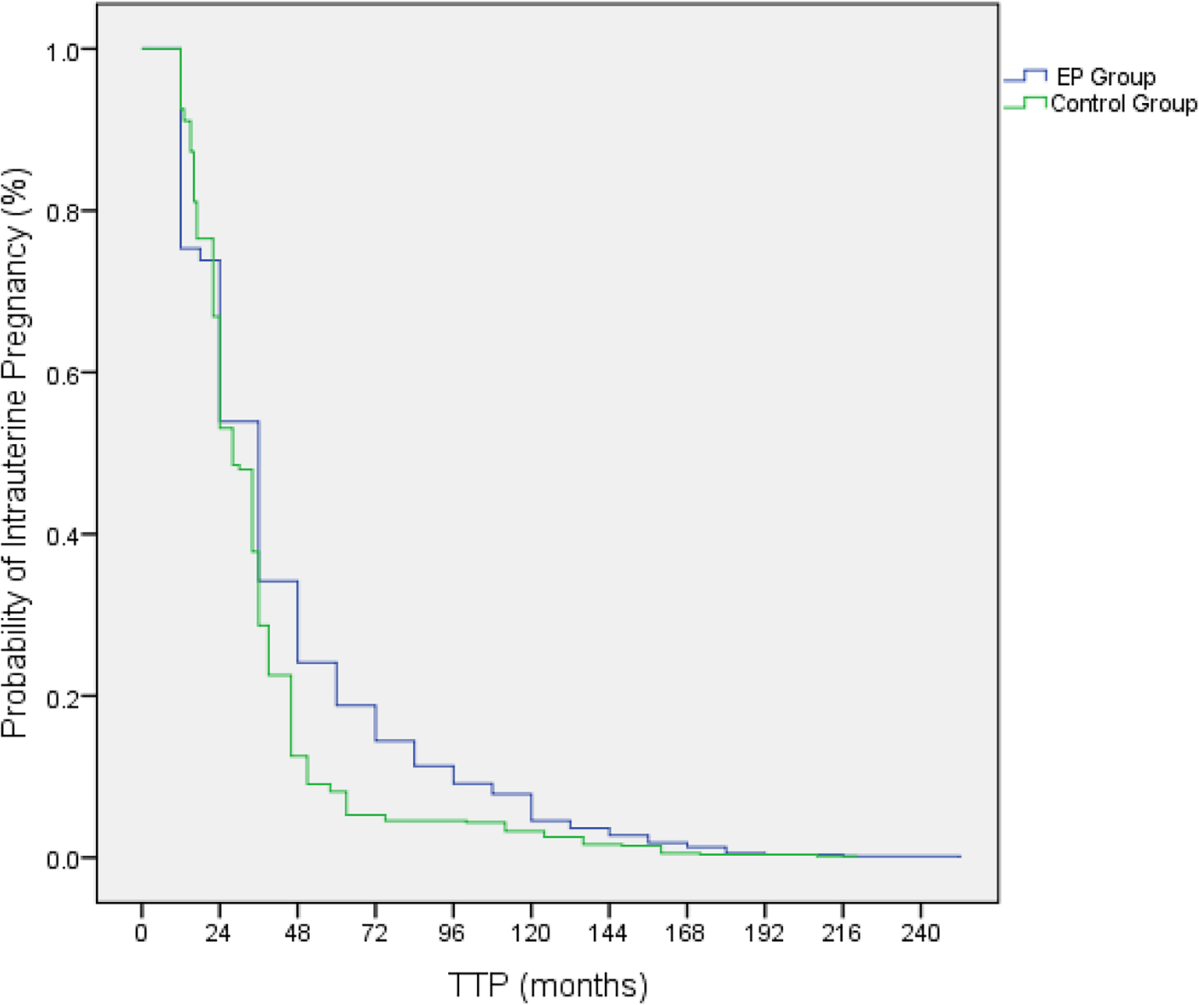
Kaplan–Meier curves for time to pregnancy in women with EP and the control. Log-rank (Mantel–Cox) comparison: P < 0.001.

Figure 1 shows the probability of intrauterine pregnancy at different TTP for women in the EP group and control group. A comparison of the curves by the Mantel–Cox log-rank test showed that a statistically significant difference could be found between the two groups (P < 0.001).

**Figure 2:**
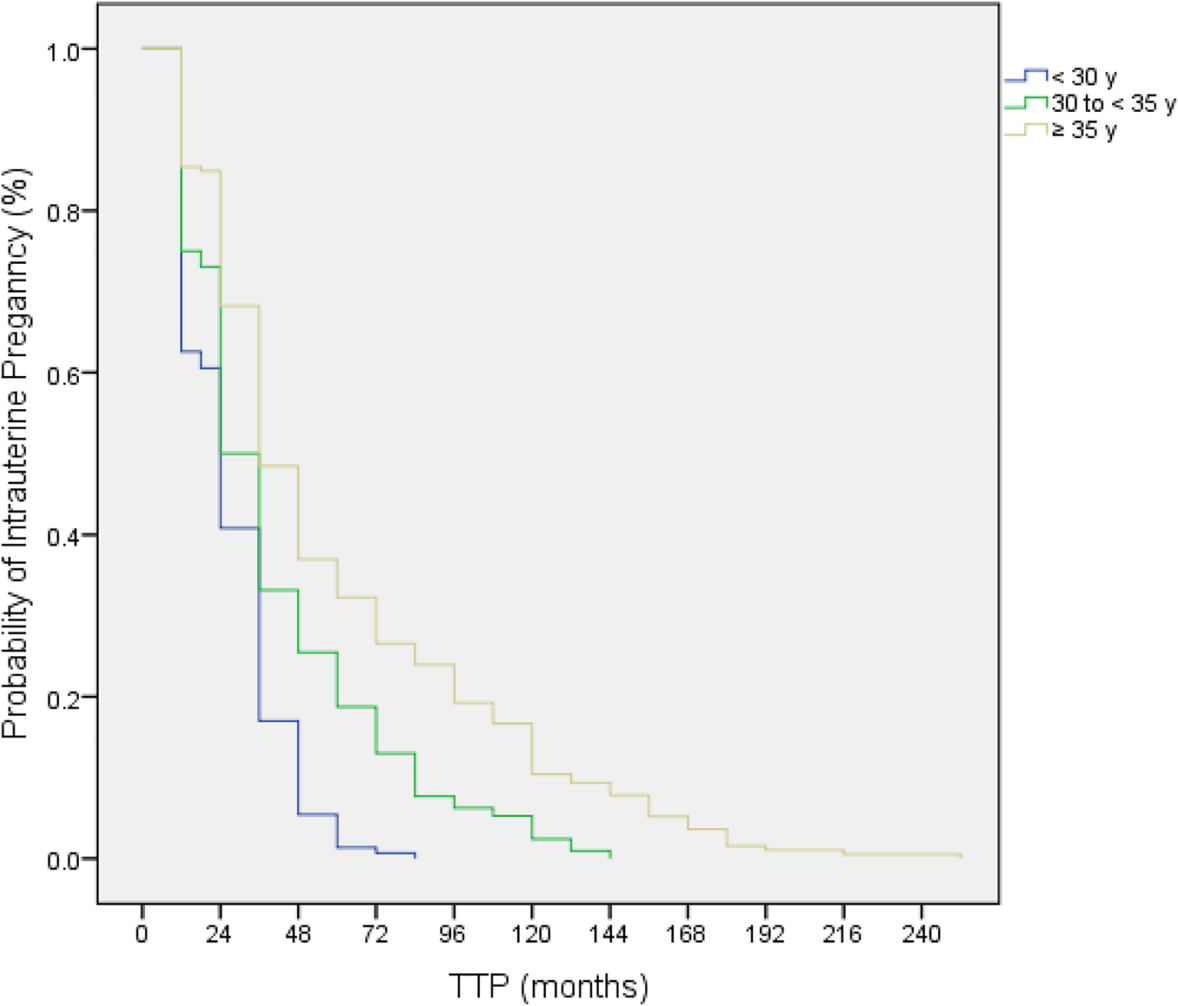
Kaplan–Meier curves for time to pregnancy in women with previous EP stratified by the maternal age. Log-rank (Mantel–Cox) comparison: P < 0.001.

The survival curves in patients with a history of EP, stratified by maternal age, were constructed by using the Kaplan–Meier method and reported in Fig. 2. There was a significant difference in the three groups (Mantel-Cox log-rank comparison: P < 0.001). In each subgroup, a decreased trend in the probability of intrauterine pregnancy was observed with the extension of TTP.

## Discussion

EP is a common and serious health problem among women of reproductive age. The factors involved with EP are still undetermined in a substantial number of women. The current evidence suggests that alterations in the tubal environment, impaired embryo-tubal transport in the fallopian tube, as well as smoking habits are negative influencing factors^21–24^. The damage to the fallopian tubes caused by previous EP may lead to reduced fecundability and is therefore one of the major reasons to undergo IVF treatment^8,9^. TTP, although easily achievable information, could become a useful clinical tool suggesting the presence of reproductive disorder in women with previous EP.

A major finding of the present study is that a significantly increased TTP was found for women with previous EP compared to women with abortion. This conclusion is supported by the finding obtained by analyzing the TTP in overall 1097 pregnancies. Moreover, the results show that, for women with previous EP, > 48 months TTP was most likely associated with low numbers of oocytes retrieved and embryos available compared to TTP of ≤ 24 months or 25–48 months, and women with younger age had a shorter TTP, higher numbers of oocytes retrieved and embryos available.

Several studies or reviews have evaluated subsequent natural conception after different EP treatments^5,6,25,26^. According to these studies, there was no statistical difference in subsequent fertility. Few studies have investigated the effect of the different treatments on the IVF outcomes^9^. Xu et al. reported that previous EP treatments including salpingectomy, salpingostomy, and conservative treatment did not statistically significantly affect IVF outcomes^9^. Consistent with the study^9^, our results showed that there is no significant difference between the three treatments.

Documents have studied that the number of oocytes retrieved positively correlated to the chance of achieving pregnancy, thus the number of oocytes retrieved was considered to be an important predictive factor for achieving pregnancy^27–29^. So, from a biological point of view, it is not surprising that an association with TTP should be found for the numbers of oocytes retrieved and embryos available in women ending in EP. For women with previous EP, longer TTP was related to a decreasing number of oocytes, which meant a reduced chance of a subsequent pregnancy. However, this finding was not found in the control group. One possible explanation is that women with previous abortion in this study had a better prognosis than those with previous EP. AFC was a marker of ovarian reserve and may be helpful in predicting the number of oocytes retrieved and stimulation response^30,31^. We collected and calculated AFC at the beginning of the cycle and found that women in the EP group had a significantly lower AFC than those in the control group. Furthermore, on the other hand, these results could also interpret the finding found in the previous studies^3,11,12^ that EP was an important cause of reproductive morbidity and had an adverse effect on subsequent reproductive success.

It is reasonable that a prolonged TTP leads to an older age at the subsequent attempt, and older age may lead to a longer TTP, which in turn may raise the risk of low fecundity. Advanced maternal age (≥ 35 years) was associated with longer TTP, lower number of oocytes retrieved and embryos available. However, these associations were weaker in younger women with < 30 and 30 to < 35 years in the present study.

The TTP was analyzed by using the survival curve constructed by the Kaplan–Meier method, which is a classical way used to evaluate the probability of pregnancy over time in a specific population. This way is generally applied to examine the infertile population. In this study, it can be successfully used for infertile women with previous EP following IVF treatment. To our knowledge, this is the first report. Enough samples were available to allow to construct reliable Kaplan–Meier survival curves for women with EP and abortion for which comparisons between the curves were performed by log-rank (Mantel–Cox) test. Also, statistical significance was observed among the different age groups. These data are consistent with those reported by Hassan and Killick who studied the effects of previous adverse reproductive outcomes on subsequent fecundity^18^. Differently from that study, the data on TTP in this study is collected from women after IVF treatment, which may be supposed to be shorter than that from natural pregnancy without infertility treatment.

The primary strength of the present study is the ability to evaluate the correlation between TTP and the number of oocytes retrieved and embryos available in women with previous EP from a single center with a large sample size. We carefully considered some confounding factors and restricted the analyses to women with a regular menstrual cycle and the same partner during the interval.

The study had a few limitations. First, because this is a retrospective study, selection bias cannot be avoided. However, considering the relatively low incidence rate of the EP in the general population, the establishment of a prospective study aimed at exploring TTP in EP would imply a follow-up of many years in a very large unselected population to pick up the women with EP, Therefore, although this research is feasible in theory, it may be very hard to be realized. Second, the causes of infertility may affect the outcome. For example, no more than 10% of cases of infertility are caused by endometrial-uterine abnormalities. These abnormalities may include endometrial polyp, submucous myoma, uterine septum, and intrauterine adhesion. In order to increase pregnancy rates, hysteroscopic surgery is routinely recommended before the IVF cycle. The duration of endometrial wound healing is different after various hysteroscopic surgeries. There is no general agreement on how long to wait for starting fresh IVF-ET cycles after surgery. As a result, the time elapsed between surgery and the start of IVF cycles may be prolonged in infertile couples. Thirdly, the type of ovarian stimulation protocols may have an effect on TTP. Ovarian stimulation is an essential part of each IVF cycle. Ovarian response largely depends on the women’s age, BMI, and reproductive history. It is difficult to make accurate predictions about the duration of stimulation for follicle maturation. Although no statistically significant difference in the proportion of ovarian stimulation protocols and the duration of stimulation between the two groups was observed, the time spent waiting for the outcome of the IVF treatment may vary in infertile couples. Finally, we cannot rule out possible unmeasured confounding as an explanation of our findings. If an unmeasured confounding factor causes couples to achieve pregnancies with a longer TTP, our associations would be biased. For instance, exposure to stress, environmental contaminants, and the disease of other systems may cause couples to have difficulty getting pregnant, but we did not have data on these variables.

In conclusion, women with previous EP have a significantly increased TTP than control women with previous abortion. For women with previous EP, > 48 months TTP is most likely associated with low numbers of oocytes retrieved and embryos available compared to TTP of ≤ 24 months or 25–48 months, and women with younger age have a shorter TTP, higher numbers of oocytes retrieved and embryos available. Indeed, EP may be caused by a range of underlying pathologies and some of the mechanisms leading to EP may play a role in the occurrence of prolonged TTP. This implies that the observed associations may be causal. Therefore, delayed TTP may serve as a useful clinical marker for identifying women with previous EP at a risk for reduced fecundability. Further study is needed to elucidate the mechanisms underlying the association between TTP and EP.

## Data Availability

All data generated or analyzed during this study are included in this published article. The datasets generated during and/or analyzed during the current study are available from the corresponding author on reasonable request.
